# Mobile phones are hazardous microbial platforms warranting robust public health and biosecurity protocols

**DOI:** 10.1038/s41598-022-14118-9

**Published:** 2022-06-15

**Authors:** Matthew Olsen, Rania Nassar, Abiola Senok, Susan Moloney, Anna Lohning, Peter Jones, Gary Grant, Mark Morgan, Dinesh Palipana, Simon McKirdy, Rashed Alghafri, Lotti Tajouri

**Affiliations:** 1 Faculty of Health Sciences and Medicine, Genomics and Molecular Biology, Bond University, Robina, Gold Coast, QLD 4229 Australia; 2 Dubai Police Scientists Council, Dubai Police, Dubai, UAE; 3 Mohammed Bin Rashid University of Medicine and Health Sciences, Dubai, UAE; 4 Oral and Biomedical Sciences, School of Dentistry, College of Biomedical and Life Sciences, Cardiff University, Cardiff, UK; 5 School of Pharmacy and Medical Sciences, Griffith University, Gold Coast, Australia; 6 School of Medicine Gold Coast, Griffith University, Gold Coast, Australia; 7 Harry Butler Institute, Murdoch University, Murdoch, WA 6150 Australia; 8 Department of Paediatrics, Gold Coast University Hospital, Southport, Australia; 9 General Department of Forensic Sciences and Criminology, Dubai Police, Dubai, UAE

**Keywords:** Public health, Risk factors

## Abstract

Advancements in technology and communication have revolutionised the twenty-first century with the introduction of mobile phones and smartphones. These phones are known to be platforms harbouring microbes with recent research shedding light on the abundance and broad spectrum of organisms they harbour. Mobile phone use in the community and in professional sectors including health care settings is a potential source of microbial dissemination. To identify the diversity of microbial genetic signature present on mobile phones owned by hospital medical staff. Twenty-six mobile phones of health care staff were swabbed. DNA extraction for downstream next generation sequencing shotgun metagenomic microbial profiling was performed. Survey questionnaires were handed to the staff to collect information on mobile phone usage and users’ behaviours. Each of the 26 mobile phones of this study was contaminated with microbes with the detection of antibiotic resistance and virulent factors. Taken together the sum of microbes and genes added together across all 26 mobile phones totalised 11,163 organisms (5714 bacteria, 675 fungi, 93 protists, 228 viruses, 4453 bacteriophages) and 2096 genes coding for antibiotic resistance and virulent factors. The survey of medical staff showed that 46% (12/26) of the participants used their mobile phones in the bathroom. Mobile phones are vectors of microbes and can contribute to microbial dissemination and nosocomial diseases worldwide. As fomites, mobile phones that are not decontaminated may pose serious risks for public health and biosecurity.

## Introduction

Mobile phones are ubiquitous and are used as primary communication devices. There are accounting for over 5 billion mobile phone users globally (over two-thirds of the world’s population) with an increase of 100 million unique mobile phone users each year^1^. According to Statista in 2020, the number of mobile phone users accessing popular messaging apps to communicate was 2.77 billion^2^. There have been many risks identified linked to the use of mobile phones including addiction^3^, vision impairment in children^4^, dangerous driving^5^, distracted pedestrians^6^, psychological stress and general anxiety^7^. Mobile phones are used up to 3 h and 37 min per person and touched with hands more than 2000 times a day^8^. A previously underestimated risk of mobile phones is associated with their role as fomite and several recent studies have confirmed the presence of viable microbes on their surface^9,10^. The United States Centre for Disease Control and Prevention (CDC) outlined that up to 80% of all infectious diseases was transmitted via hands^11^. Researchers have shown that mobile phones are reservoirs of microbes, users neglect and rarely decontaminate these devices, high rates of use and touch contact of mobile phone surfaces and individual’ tendencies to touch their face regularly (up to 23 times an hour)^12^ or/and other surrounding surfaces^13^. Olsen et al. (2020) stated that mobile phones act as ‘Trojan horse’ devices which: (i) bypass gold standard hand hygiene practices; (ii) are likely linked to pathogen movement via cross-contamination transmission pathways during epidemics and pandemics^14^; and (iii) contribute to global population infections and hospitalisations due to nosocomial infections. A recently published survey of 165 healthcare workers (HCW) demonstrated that 52% (86/165) of participants used their mobile phone in the bathroom/toilet and that 57% reported that they never wash their devices^15^. Mobile phones are platforms that host microbial vectors leading to the dissemination of infectious diseases. The use of phones by all professional sectors makes them ideal platform niches for micro-organism contamination^10^. Despite a massive increase in published articles describing the role of mobile phones as fomites there is still poor global awareness with continuing poor practices of standardised sanitisation. In 2020, a global scoping review of 56 studies identified that on average, 68% of mobile phones were contaminated with microbes with many harbouring antibiotic resistant bacteria^9^. While such scoping review was informative, microbial characterisation and identification from these studies most probably underestimated the overall spectrum and richness of microbes on mobile phones. These studies were based on traditional agar-based growths, biochemical testings or orthogonal polymerase chain reaction amplification of microbial genomic sequences^9^.

Improved methodology using a sequencing approach with 16S rRNA primers for metagenomic sequencing also highlighted the inadequacy of traditional culture-dependent identification techniques to capture the entire globality of microbes present on mobile phones^16^. A 2021 pilot next generation sequencing project was able to capture a wider population of micro-organisms with all mobile phones found to be contaminated with microbes. The findings consisted of 235 bacteria, 8 fungi, 8 protists and 53 bacteriophages reported from only five mobile phones derived swabs^14^. However, this study still could be considered as an underestimation of microbial finding as the samples were pre-cultured on agar plates prior to next generation sequencing metagenomic profiling^14^. A similar study used a metagenomic shotgun sequencing-based approach of viable pre-cultured microbes collected from 30 mobile phones of HCW. These phones were swabbed across 4 different hospital wards and plated on five different agar plates (Horse Blood agar, Nutrient agar, MacConkey agar, Bile Esculin agar, Mannitol Salt agar) before being subject to next generation sequencing^10^. The study identified a large range of microbial organisms with 399 operational taxonomic units (OTUs) bacteria, 155 bacteriophage OTUs and the identification of 134 antibiotic resistant genes (ARGs) and 347 virulence factor genes (VFGs).

To address the limitations identified in previous studies, this study collected swabs from mobile phones of health care staff working in a hospital. These swabs were subject to a direct shotgun next generation sequencing to identify the metagenomic presence of micro-organisms on these surfaces.

## Methods

### Participant recruitment and sample collection

Informed consent was obtained from all subjects of this study with a total of 26 health care workers from the Paediatric Intensive Care Unit (PICU) and the General Paediatric Department (GPD) of the Gold Coast University Hospital, Australia. An information sheet was provided to all participants, detailing the nature of the research, with no personal identifying information collected. Informed consent was provided verbally and agreeing to participate on the day of sampling. Samples were collected each of the 26 mobile phones using culture swab EZ II swabs (Becton Dickson) pre-moistened with sterile saline. During the sample collection phase, gloves were worn and changed between participants to prevent cross-contamination. The mobile phones were swabbed on both the front and back of the devices with swabs then placed in portable containers and transported immediately to the laboratory for processing.

### Survey questionnaire

The complete survey data set has been published previously^15^, however, for this paper some results have been extracted to enable comparison with the microorganisms discovered. The 26 questionnaire survey responses were included in this paper.

### Swab and DNA extraction

The preliminary step of the DNA extraction process involved the use of bead beating with 0.1 mm diameter glass beads (BioSpec Products, Bartlesville, OK USA) on a Powerlyser 24 homogenizer (Mo-Bio, Carlsbad, CA USA) at the Australian Centre for Ecogenomics (ACE), Brisbane, Australia. Briefly, samples were transferred to a bead tube and 800 µl of bead solution (Qiagen, Germantown, MD USA) was added and bead-beat for five minutes at 2000 rpm, then centrifuged at 10,000 g for one minute. Following the addition of 60 µl of cell lysis buffer, tubes were vortexed and then heated at 65 °C for 10 min (while mixing at 1000 rpm), then vortexed again for 30 s and stored at −20 °C pending DNA extraction. Prior to DNA extraction, samples were thawed at room temperature; vortexed and centrifuged for one minute at 10,000 g. The resulting lysate was transferred to a new collection tube and DNA extraction carried out using DNeasy Powersoil Kit (Qiagen), as per manufacturer protocol with a final elution volume of 50 µl using sterile, EDTA-free elution buffer.

### Metagenomic sequencing and bioinformatic analysis

Libraries were prepared according to the manufacturer’s protocol using Nextera DNA Flex Library Preparation Kit (Illumina San Diego, CA USA). Preparation and bead clean-up were run on the Mantis Liquid Handler (Formulatrix) and Epmotion (Eppendorf) automated platform. On completion of the library prep protocol, each library was quantified, and quality control (QC) was performed using the Quant-iT™ dsDNA HS Assay Kit (Invitrogen, Carlsbad, CA USA) and Agilent D1000 HS tapes on the TapeStation 4200 (Agilent Technologies, Santa Clara, CA USA) as per manufacturer’s protocol. Library Pooling, QC and Loading Nextera DNA Flex libraries were pooled at equimolar amounts of 2 nM per library to create a sequencing pool. The library pool was quantified in triplicates using the Qubit™ dsDNA HS Assay Kit (Invitrogen). Sequencing was carried out on the NextSeq500 (Illumina) using NextSeq 500/550 High Output v2 2 × 150 bp paired end chemistry according to manufacturer’s protocol^12^. The post-sequencing derived raw data were retained and transferred into Illumina base space platform (https://basespace.illumina.com). Following the sequencing runs, data as demultiplexed FASTQ files were uploaded into CosmosID platform (https://www.cosmosid.com/). Raw datasets Fastq files were analysed using the CosmosID software to identify bacteria, fungi, virulence factor genes and antibiotic resistance genes. The datasets were then analysed with proper mining bio-informatic analytic tools using high performance data-mining k-mer algorithm and highly dynamic comparator databases (GenBook®). Through this process, the raw data of millions of short reads can be distinctively aligned against sequences of microbial genomes and genes (CosmosID Metagenomics Cloud).

Microbial ‘Richness’ corresponds to the cumulative amount of all distinct microbes detected across all phones whereas the number of occurrences across all phones for each of these distinct microbes are represented by Hits.

### Ethics

Ethical approval was obtained from Bond University Human Research Ethics Committee (16,004) and the GCUH Human Research Ethics committee with Site Specific approval (GC HREA 46,569). All methods were performed in accordance with the relevant guidelines and regulations.

## Results

### Participant features and questionnaire findings

In total, there were 26 health care workers who participated in this study: 16 nurses, 8 doctors, 1 outpatient clinical staff and 1 unspecified participant. 16 staff members were from the General Paediatric Department and 10 were from the Paediatric Intensive Care Unit. Majority of the participants (77%; N = 20/26) were completing their shift and whilst 23% (n/N = 6/26) commencing their shift. 77% (20/26) reported using their mobile phones at work with 88% (23/26) believing their mobile phones were essential tools for their job. 96% (25/26) of participants believed their mobile phones would harbour potentially pathogenic microorganisms. Concerning the hygiene habits associated with mobile phone use in the professional setting, 46% (12/26) of the participants had recently used their mobile phones in the bathroom. Of the medical staff using mobile phones in the bathroom, 58% (7/12) reported using their devices for social media access, 25% (3/12) did not specify the purpose of use and 16% (2/12) reported using their phone for work-related purposes. Over half of the participants (54%; n/N = 14/26) of participants had never cleaned their mobile phone. Of the 46% (12/26) of participants who had cleaned their mobile phones at some point, 25% (3/12) did so within the past year, 33% (4/12) did so within the past month, 16% (2/12) did so within the past week and 25% (3/12) did so within the past day. Of those who reported cleaning their phones, 41% (5/12) used an alcohol-based wipe and 33% (4/12) used a disinfectant spray.

### Illumina derived next generation sequencing datasets

#### Reads

The average amount of sequencing reads per mobile phone was approximately 53 million reads. Sample 26 (NS313-110) contained the lowest (33 million) and sample 12 (NS250-72) the highest number of reads (156 million) respectively.

The sequencing fastq dataset files of all sequencing samples of this study are available and processed in the SRA database with the SRA BioProject accession number PRJNA828402 that can be available in Entrez (https://www.ncbi.nlm.nih.gov/sra/PRJNA828402). Each detailed accession number of the 26 datasets generated and analysed during the current study are available in the NCBI repository, (PRJNA828402—SRA—NCBI(nih.gov)).”

#### Sequencing reads and metagenomic overview

A total of 11,163 microorganisms and 2096 genes coding for antibiotic and virulent factors were identified in this metagenomic shotgun next generation sequencing study. In total, there were 5714 bacteria, 675 fungi, 93 protists, 228 viruses, 4453 bacteriophages, 560 antibiotic resistant genes and 1 536 virulence factor genes identified across the 26 mobile phones from GPD and PICU (Table 1).

**Table 1:** Number of all microorganisms and genes found on each mobile phone (per ward) via shotgun-metagenomic sequencing.

Ward	Occupation	Sample	Code	Number of identified Microorganisms/AR Genes and VF Genes Discovered						
				Bacteria	Fungi	Protists	Viruses	Bacteriophages	AR Genes	VF Genes
GPD	Unspecified	Sample 1	NS231-02	197	26	5	3	148	20	93
GPD	Ward nurse	Sample 2	NS231-03	227	32	2	9	204	21	49
GPD	Ward doctor	Sample 3	NS231-06	143	26	2	7	179	6	12
GPD	Ward doctor	Sample 4	NS231-07	301	33	7	12	205	29	81
GPD	Ward nurse	Sample 5	NS231-08	197	24	3	9	203	16	57
GPD	Ward nurse	Sample 6	NS231-09	229	24	6	14	195	23	48
GPD	Ward nurse	Sample 7	NS231-12	270	17	2	23	180	15	44
GPD	Ward nurse	Sample 8	NS231-14	189	34	5	4	185	26	61
GPD	Ward doctor	Sample 9	NS231-15	176	26	2	13	192	16	39
GPD	Ward doctor	Sample 10	NS231-16	170	24	6	8	162	28	88
GPD	Ward doctor	Sample 11	NS231-19	337	42	6	10	231	47	97
GPD	Ward nurse	Sample 12	NS250-72	246	27	5	5	156	22	66
GPD	Ward nurse	Sample 13	NS250-73	316	39	6	20	235	28	61
GPD	Ward nurse	Sample 14	NS251-34	280	34	6	15	228	34	57
GPD	Ward nurse	Sample 15	R6298_S36	287	25	3	14	210	17	49
GPD	Ward nurse	Sample 16	R6301_S37	197	27	3	11	189	19	55
PICU	Ward doctor	Sample 17	NS300_02	200	20	3	9	163	30	51
PICU	Ward nurse	Sample 18	NS300_05	213	22	1	9	158	14	28
PICU	Ward nurse	Sample 19	NS300_06	105	4	1	0	54	12	74
PICU	Ward doctor	Sample 20	NS300_07	59	7	0	3	68	2	5
PICU	Ward doctor	Sample 21	NS300_09	216	22	3	4	111	16	66
PICU	Ward nurse	Sample 22	NS300_10	371	52	8	14	221	34	101
PICU	Ward nurse	Sample 23	NS300_11	256	39	2	4	174	30	83
PICU	Ward nurse	Sample 24	NS312-54	203	21	2	2	149	15	34
PICU	Ward nurse	Sample 25	NS312-56	187	13	1	4	133	14	53
PICU	Outpatient Clinical Staff	Sample 26	NS313-110	142	15	3	2	120	26	84
Total				5714	675	93	228	4453	560	1536

AR = Antibiotic resistance; VF = Virulence factors.

**Figure 1:**
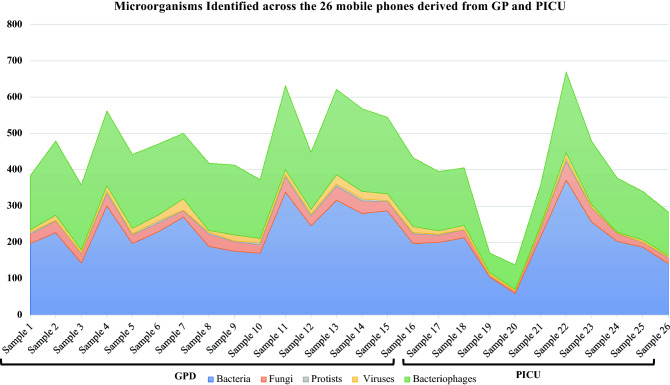
Distribution of different types of microorganisms across the 26 mobile phone samples.

On average, mobile phones from the GPD contained higher amounts of pathogens and genes, compared to the phones sampled from PICU. Additionally, mobile phones of nurses contained in average a slightly higher number of microbes compared to doctors with 460.2 and 403.6 respectively. Across all 26 mobile phones, the average number of micro-organisms was calculated to be 429 with an average of 477.7 on the GPD phones and 361.6 in the PICU phones. Microbial numbers ranged from 138 to 669 per phone and genes (ARG and VRG) ranged from 7 to 144 per phone. (Table 1). Bacteria and bacteriophages represented the largest proportion of the microorganism distribution (Fig. 1).

##### Bacterial identification

1307 bacterial different strains were found with a richness across all 26 mobile phones accounting for 5714 hits. Clinically relevant species were found and include bacteria responsible for nosocomial diseases. 143 ‘ESKAPE’ type bacteria were found and consisted of Enterobacteriaceae: [46 hits on 19 phones (73%; 19/26) ], *Staphylococcus aureus* [25 hits; 25 phones (96%; 25/26)], *Klebsiella pneumoniae* [2 hits; 2 phones (7.7%; 2/26)], *Acinetobacter baumannii* [33 hits; 22 mobile phones (84.6%; 22/26)], *Pseudomonas aeruginosa* [21 hits, 21 mobile phones (80.8%; 21/26], *Enterococcus faecalis*/*E. faecium* [14 hits; with 50% of all 26 phones contaminated]. Of note, different strains of *Pseudomonas* and *Acinetobacter* species accounted for 187 and 205 richness hits respectively across the 26 mobile phones.

Additionally, community-acquired pathogenic HACEK group gram-negative bacteria accounted for 180 richness hits across the mobile phones swabbed. The highest hits were attributed to *Haemophilus* spp, and *Aggregatibacter* spp with 110 and 38 hits respectively while *Cardiobacterium hominis*, *Eikenella corrodens*, and *Kingella* spp corresponded to 14, 12 and 6 hits respectively. Every single phone swab harboured at least one *Haemophilus* spp.

Coagulase negative staphylococci (CONS) was found on all the mobile phones accounting for a total of 272 richness hits. All phones within that study harboured CONS with *S. epidermidis*, *S. hominis*, *S. warneri*., *S. haemolyticus. S. lugdunensis* was identified on 92% (24/26) of mobile phones. *While S. capitis* and *S. pasteuri* in 88% and *81%* of phones respectively.

*Neisseria* spp were identified with 152 richness hits. *N. flavescens*, *N. subflava*, *N. elongate, N. sicca*, and *N. mucosa* were the most represented with 21, 16, 16, 16 and 14 hits respectively. Noteworthy, *N. meningitidis* were present on 27% of phones (7/26) and *N. gonorrhoeae* was retrieved from one phone.

**Figure 2:**
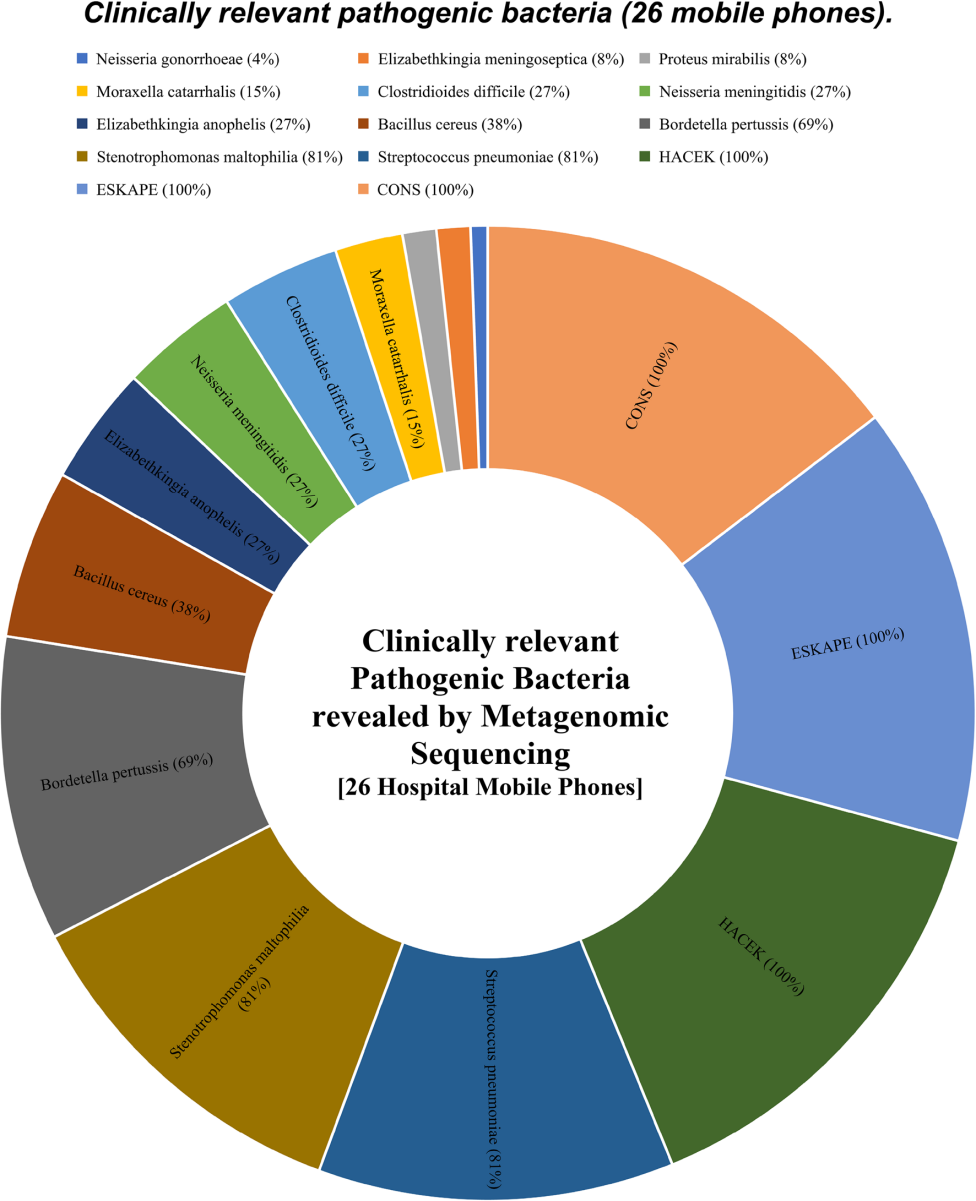
Clinically relevant pathogenic bacteria identified across all 26 mobile phones.

Streptococci strains accounted for 404 richness hits across the 26 mobile phones and included *S. thermophilus*, *S. sanguninis*, *S. parasanguinis*, *S. salivarius*, *S. pseudopnemoniae*, *S. oralis*, *S. mitis*, *S. intermedius, S. infantis*, *S. infantarius*, *S. cristatus*, *S. australis*, *S. anginosus*, and *S. agalactiae*. *S. pneumoniae* was found on the surface of 81% of the mobile phones (21/26) (Fig. 2).

**Figure 3:**
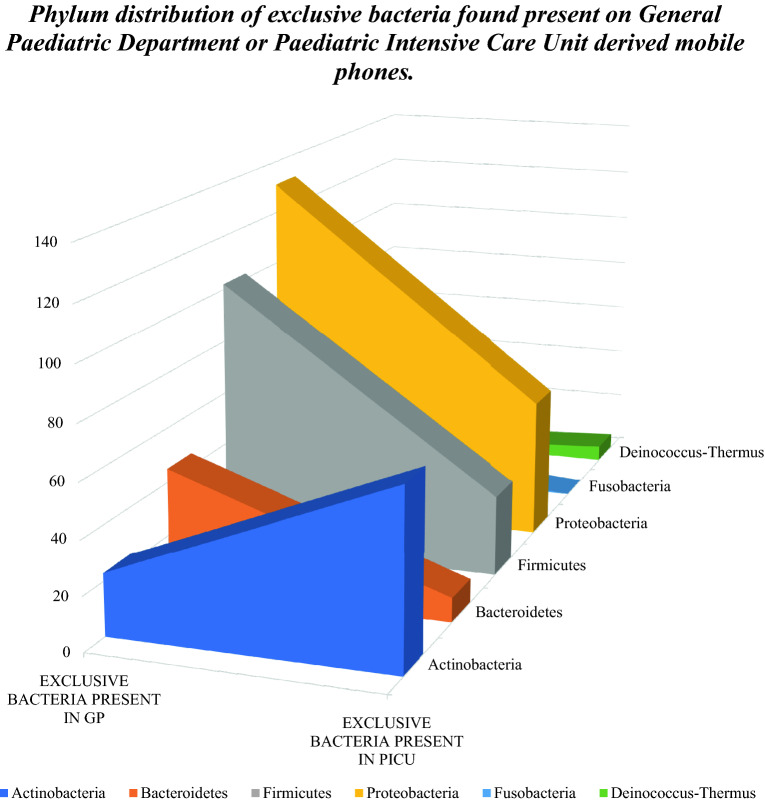
Phylum distribution of exclusive bacteria found present on General Paediatric Department or Paediatric Intensive Care Unit derived mobile phones.

Mobile phones microbial composition varied with a subset of microbes uniquely present in either department: 170 and 317 bacteria in PICU and GPD respectively. These unique ward bacterial signatures showed different bacterial phylum profiles with the bacterial *Actinobacteria* phylum demonstrating the larger signature subset of PICU derived mobile phones while *Bacteroidetes*, *Firmicutes*, and *Proteobacteria* phylum were predominant in GPD derived devices (Fig. 3).

##### Bacteriophage identification

**Figure 4:**
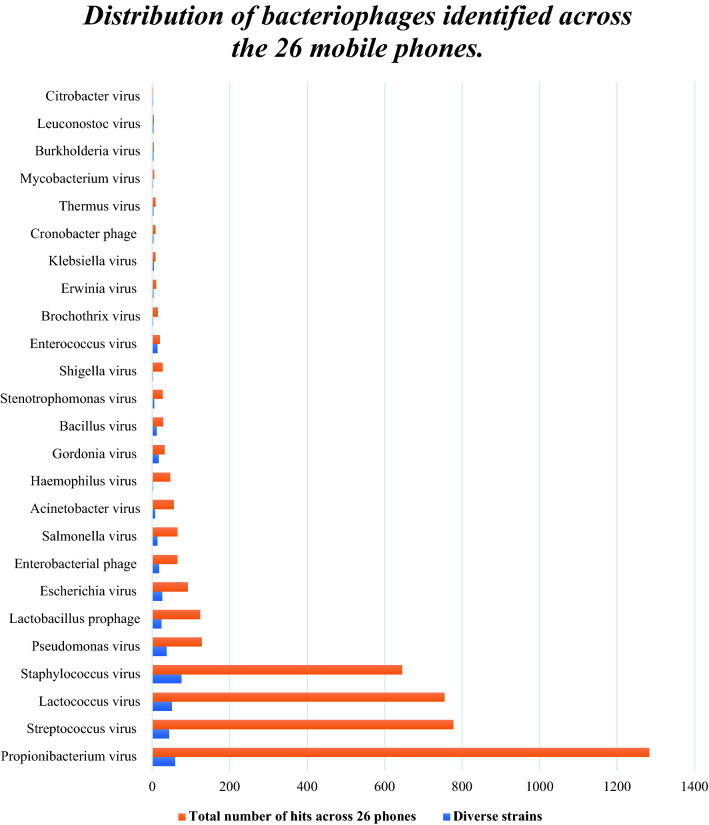
Distribution of bacteriophages identified across the 26 mobile phones.

In total there were 512 different bacteriophage viruses accounting for 4453 hits. Figure 4 illustrates the various bacteriophages identified from mobile phones of the GPD and PICU hospital departments. The highest hits corresponded to *Propionibacterium virus*, *Streptococcus virus*, *Lactococcus virus*, *Staphylococcus virus, Pseudomonas virus* with 29% (1 283/4 453), ~ 17.5% (777/4 453), ~ 17% (755/4 453), ~ 14.5% (646/4 453), ~ 3% (128/4 453) respectively (Fig. 4).

**Figure 5:**
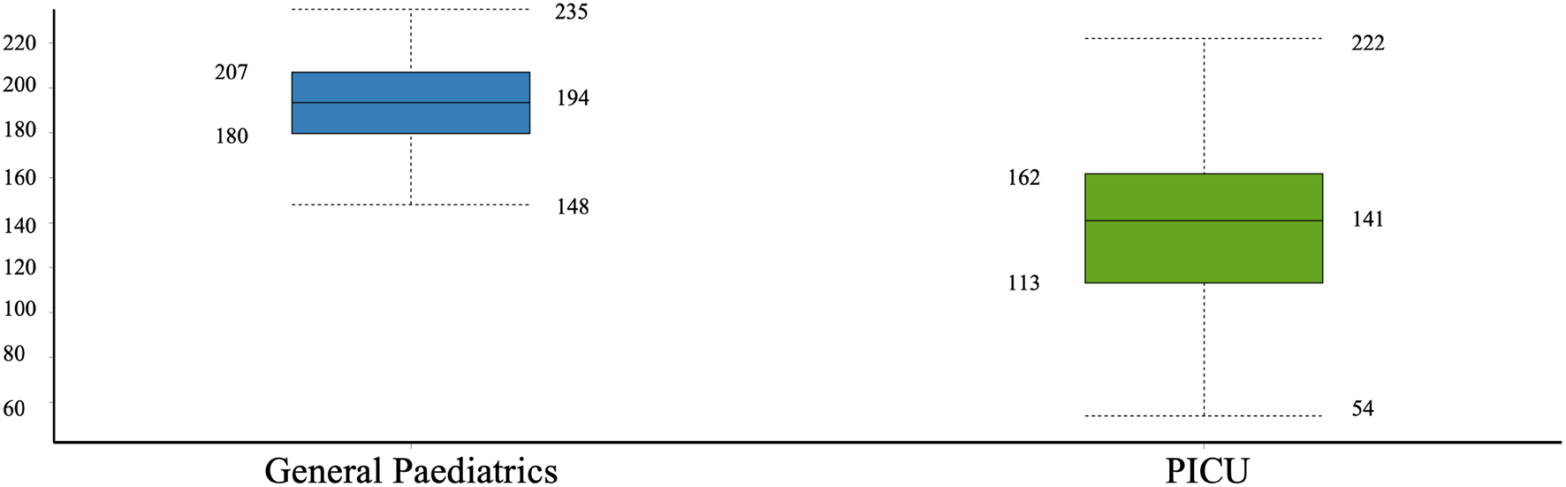
Boxplot of bacteriophages in GPD versus PICU wards (CHAO1 representation).

A significant difference in the number of bacteriophages was observed between the two wards (GPD and PICU) (*P*-value: 0.0022) (Wilcoxon Rank Sum Test) (Fig. 5).

##### Viral identification

Sixty-seven different viruses accounting for 228 richness hits was found on the mobile phones. Seven different human herpes viruses (HHV) were identified and corresponded to a total richness of 29 hits. 15 phones had at least one HHV and in one phone alone 5 HHVs could be retrieved [*Herpes Simplex virus 1*, *Epstein bar Virus*, *cytomegalovirus*, *Roseolovirus 6* and *7*]. Twenty-nine different strains of *Human papillomavirus* were found which corresponded to 95 total hit richness across the mobile phones swabbed in this experiment. Seven pathogenic Human Papilloma Viruses (HPVs) (24%;7/29) were present and these accounted for 45% (43/95 hits) of all the 95 HPV hits. Of note, one phone alone had 5 pathogenic HPVs (HPV-3, -4, -5, -9 and -49). Polyomaviruses such as the *Human polyomavirus 6*, *MW* and *STL polyomavirus* were identified. Noteworthy, the *Merkel cell polyomavirus* was retrieved on six mobile phones.

##### Protist identification

**Figure 6:**
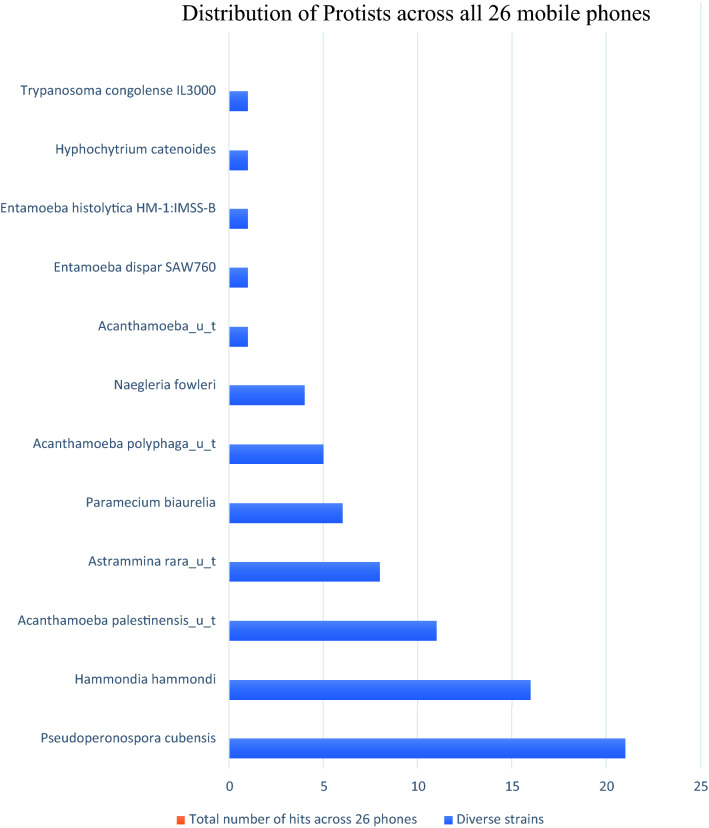
Distribution of protists identified across 26 mobile phones.

12 different protists were found representing 93 total hits. Figure 6 highlights the range of protozoa identified with several amoebae of the protozoal group Sarcodina with *Acanthamoeba polyphaga*, *Acanthamoeba palestinensis*, *Naegleria fowleri*, *Entamoeba dispar*, *Entamoeba histolytica* (Fig. 6).

#### Resistome and virulome

##### Antibiotic resistance genes

**Figure 7:**
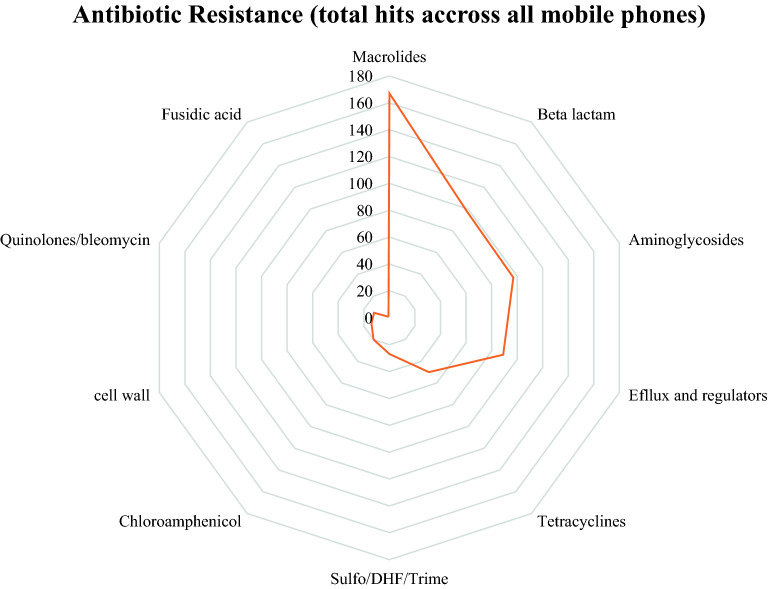
Antibiotic Resistant gene distribution across all wards of 26 mobile phones.

**Figure 8:**
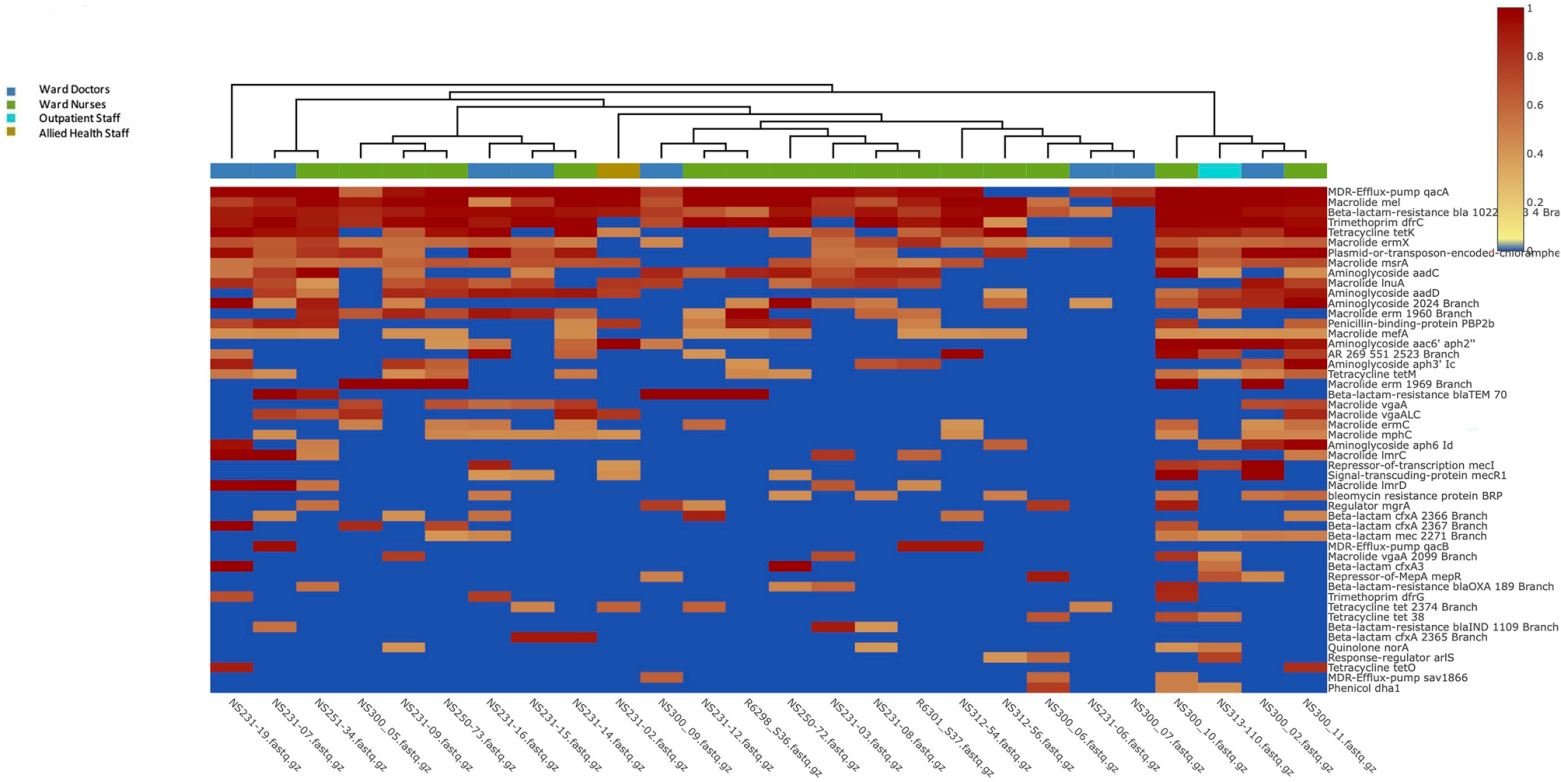
Heatmap representation of antibiotic resistant genes found on mobile phones owned by health care staff (heatmap clustered by staff occupation).

The metagenomic analysis revealed the presence of 134 different (distinct) antibiotic resistance genes with a cumulative richness number across all the mobile phones of 560 ARGs. Figure 7 represents the distribution of grouped antibiotic resistant genes. Resistance genes to Macrolides (19 genes), beta-lactams (32 genes), aminoglycosides (26 genes), and tetracycline (13 genes) corresponded to richness hits of 167, 98, 97 and 50 respectively (Fig. 7). Multi-type of antibiotics was targeted by efflux pumps (17 genes) and pump-regulator genes (13 genes) which together accounted for 89 richness hits. Less richness was found for other antibiotics resistance genes acting on bacterial metabolism (sul2 gene acting on Sulphonamides; dfrC and dfrG genes acting on Trimethoprim), on cell wall (PBP1b/2b and vanXY genes acting on transpeptidases and vancomycin), on bacterial DNA (norA, oqxA, bleomycin binding protein genes) and on protein translation [genes like cmx, dha1, cm acting on phenicols; fusC gene acting on the bacterial elongation factor (EF)] Fig. 8 (and Supplementary Fig. 1).

##### *Virulence factor genes* (VFGs)

**Figure 9:**
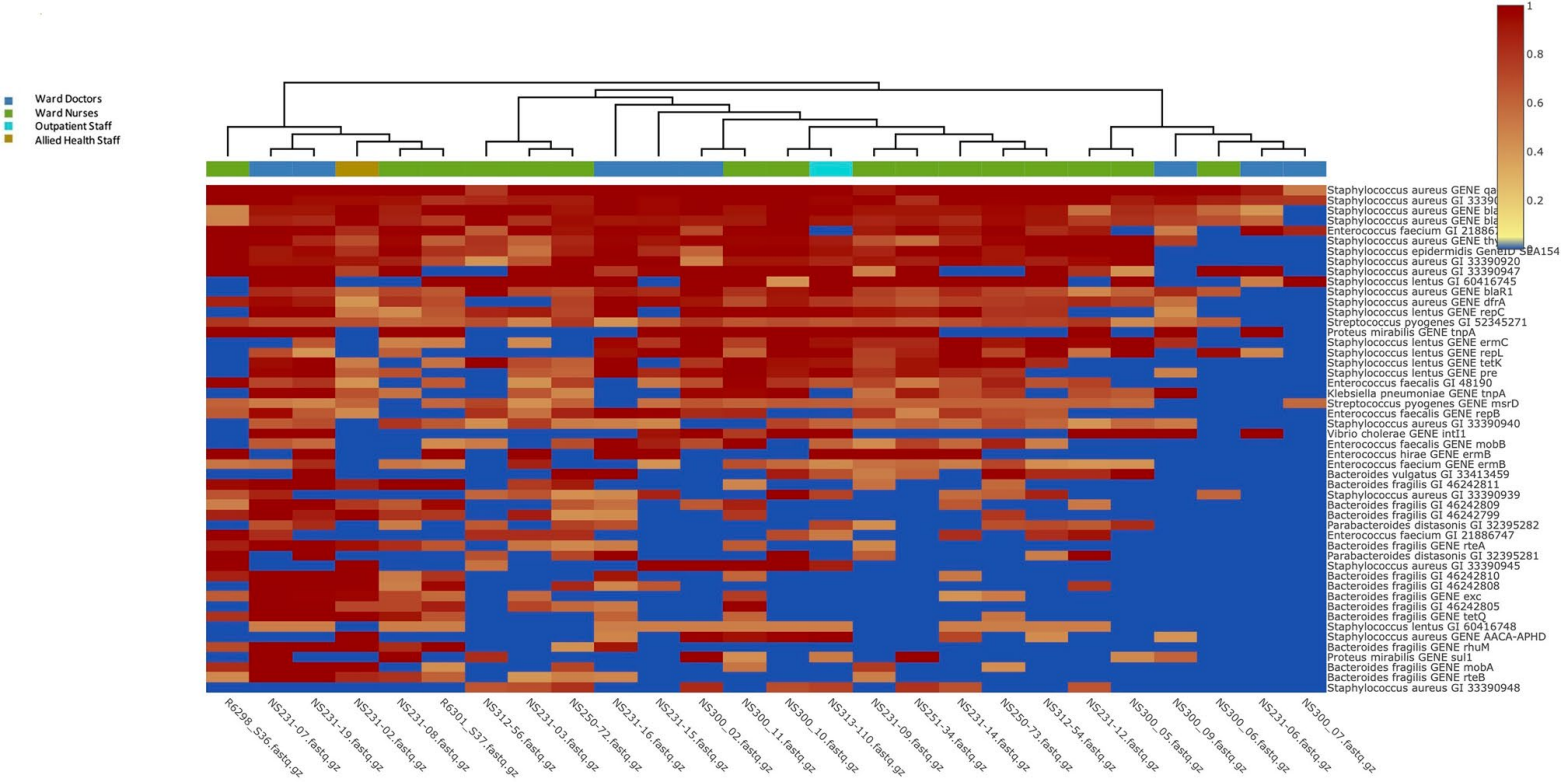
Heatmap representation by healthcare occupation of the 419 distinct virulence factor genes identified on mobile phones by means of metagenomic analysis.

Across the mobile phones swabbed, this study identified 419 different (distinct) virulent factor genes with 1536 hits. 35% of all these hits (552/1536) were attributed to 28 different VFGs genes that were all in at least 50% of mobile phones and included *Klebsiella pneumoniae* GENE tnpA, *Proteus mirabilis* GENE tnpA, *Enterococcus faecalis* GENE repB & GENE mob, *Enterococcus faecium* GENE ermB, *Streptococcus pyogenes* GENE msrD, *Staphylococcus epidermidis* GeneID SEA1545, *Staphylococcus lentus* GENE tetK & GENE repL & GENE repC & GENE pre & GENE ermC, *Staphylococcus aureus* GENE qacC & GENE dfrA & GENE blaZ & GENE blaR1 & GENE blaI & GENE thyA (Fig. 9 and Supplementary Fig. 2).

## Discussion

This study performed metagenomic profiling of swabs derived from 26 mobile phones of health care workers, predominantly doctors and nurses, in a Paediatric Intensive Care Unit and a General Paediatric Department). Alongside the shotgun next generation sequencing experimentation, a questionnaire was competed by all participants. Results showed that all phones were contaminated with microbes including bacteria, viruses, fungi and protozoa. The average microbial burden on the mobile phone showed that phones derived from GPD had the greatest number of bacteria, fungi, viruses and protists with 235, 29, 15 and 4 micro-organism respectively. Mobile phones from the PICU harboured on average 195 bacteria, 22 fungi, 7 viruses. Interestingly average number of bacteriophages were also more common on mobile phones from the GPD versus PICU with 194 and 141 respectively (Fig. 5). This ward microbial burden difference was observed in both nursing and medical staff. The reduction of mobile phone microbial burden in PICU might be associated with higher frequency of hand hygiene practices or more stringent infection control measures. Interestingly, the average number of microbes irrespective of the ward was always higher in mobile phones owned by nurses than doctors with the exception of fungi and protists that were found in higher number on doctor phones from the GPD. Additionally, mobile phones of the doctors from the GPD had a higher number of antibiotic resistant and virulent factor genes than those of nurses. However, in PICU, nurses’ mobile phones had a higher number of antibiotic resistant and virulent factor genes compared to doctors within that department. Overall, the microbial load on phones from both departments was at levels that should be considered problematic. For bacteria alone, this metagenomic analysis identified 1307 different strains accounting for 5714 hits from 26 mobile phones. Well-known nosocomial organisms including HACEK bacteria causing endocarditis [*Haemophilus* spp, *Aggregatibacter* spp *Cardiobacterium hominis*, *Eikenella corrodens*, and *Kingella* spp] and ESKAPE type bacteria [Enterobacteriaceae, *Staphylococcus aureus*, *Klebsiella pneumoniae, Acinetobacter baumannii*, *Pseudomonas aeruginosa* and *Enterococcus faecalis*/*E. faecium* organisms] were present on all 26 mobile phones swabbed in this study. This study has identified a serious general hospital infection control concern that may escalate to future public health threats.

The study also identified other microbial presence on mobile phones that raises concerns. Clinically relevant pathogens such as *Bordetella pertussis*, responsible for whooping cough was present on 69% of all phones studied, *Streptococcus pneumoniae* and the emergent nosocomial bacteria *Stenotrophomonas maltophilia* were each present on 81% of all phones studied (21/26).

Food borne bacteria (*Bacillus cereus*) was identified on HCW mobile phones. While this study was done in a hospital setting, it confirms that other industries such as the food industry are also at risk of microbial cross contamination from mobile phones. Other concerning organisms including *Clostridioides difficile, Moraxella catarrhalis, Proteus mirabilis, Elizabethkingia meningoseptica* and the sexually transmitted infectious bacteria *Neisseria gonorrhoeae* were identified on phones in this study. *Clostridioides difficile* infections has been shown to spread from contaminated surfaces with the risk of infection higher when using bathrooms preceded by infected individuals^17^. Finding HCW mobile phones to be microbial laden fomites possibly confers appropriate conditions to disseminate infections to susceptible hosts and immune-compromised patients and is a real public health risk. One example is finding *Elizabethkingi*a *meningoseptica*, a nosocomial bacterium that has disastrous consequences on premature babies with known past outbreaks linked to phone receivers^18^.

Human behaviours and the constant contact with mobile phones in toilets provide cumulative evidence that such devices are exposed to unsanitary conditions leading to the presence of a range of viable microbes on these platforms. Based on this study and others, it appears mobile phones are rarely or ever cleaned and even when cleaned this may occur in an ineffective manner. Mobile phones act as fomites turning these devices into ideal platforms for disease transmission either by means of self-inoculation when touching your own mobile phone and face orby simple microbial dissemination in the environment, public places, or professional sectors.

Bacteriophages were also found in association with bacteria with 512 different phages found and accounting for 4453 hits across 26 HCW mobile phones. Additionally, 67 different viruses including animal and human viruses were detected. These consisted of seven different human herpes viruses with 15 phones found with at least one HHV and one phone harboured up to 5 HHVs. including *Herpes Simplex virus 1*, *Epstein bar Virus*, *cytomegalovirus* and *Roseolovirus 6* and *7*. 29 different strains of Human papillomavirus were found with seven clinically important pathogenic HPVs and Merkel *cell polyomavirus* responsible for a rare but highly aggressive form of cancer was retrieved on six mobile phones from HCW suggesting a role for transfer of significant viral infections from mobile phones.

This study has also highlighted the risk posed by the presence of a large profile of antimicrobial drug resistome and pathogenic virulome on the surface of mobile phones. The bacterial resistome found in the study showed antibiotic resistant genes that counteract with all antibiotic modes of action on bacteria. Antibiotics normally actively targeting bacterial cell wall, cell membrane, cellular metabolism, DNA transcription & replication and protein translational synthesis may be impacted by the expression of these antibiotic resistance genes. Of note, 17 genes coding for drug efflux pumps were found in this study demonstrating that the resistome capacity of bacteria present on mobile phones is equipped with sophisticated expulsion processes protecting them from ‘undesirable’ antibiotics.

Along with the antibiotic resistome profile, the bacteria found on mobile phones show strong virulence capacity with 419 different virulent factor identified genes (1536 hits across all 26 mobile phones). High amount of VFGs were the signature of *Klebsiella pneumoniae*, *Proteus mirabilis*, *Enterococcus faecalis*, *Enterococcus faecium*, *Streptococcus pyogenes*, *Staphylococcus epidermidis*, *Staphylococcus lentus*, *Staphylococcus aureus*.

**Figure 10:**
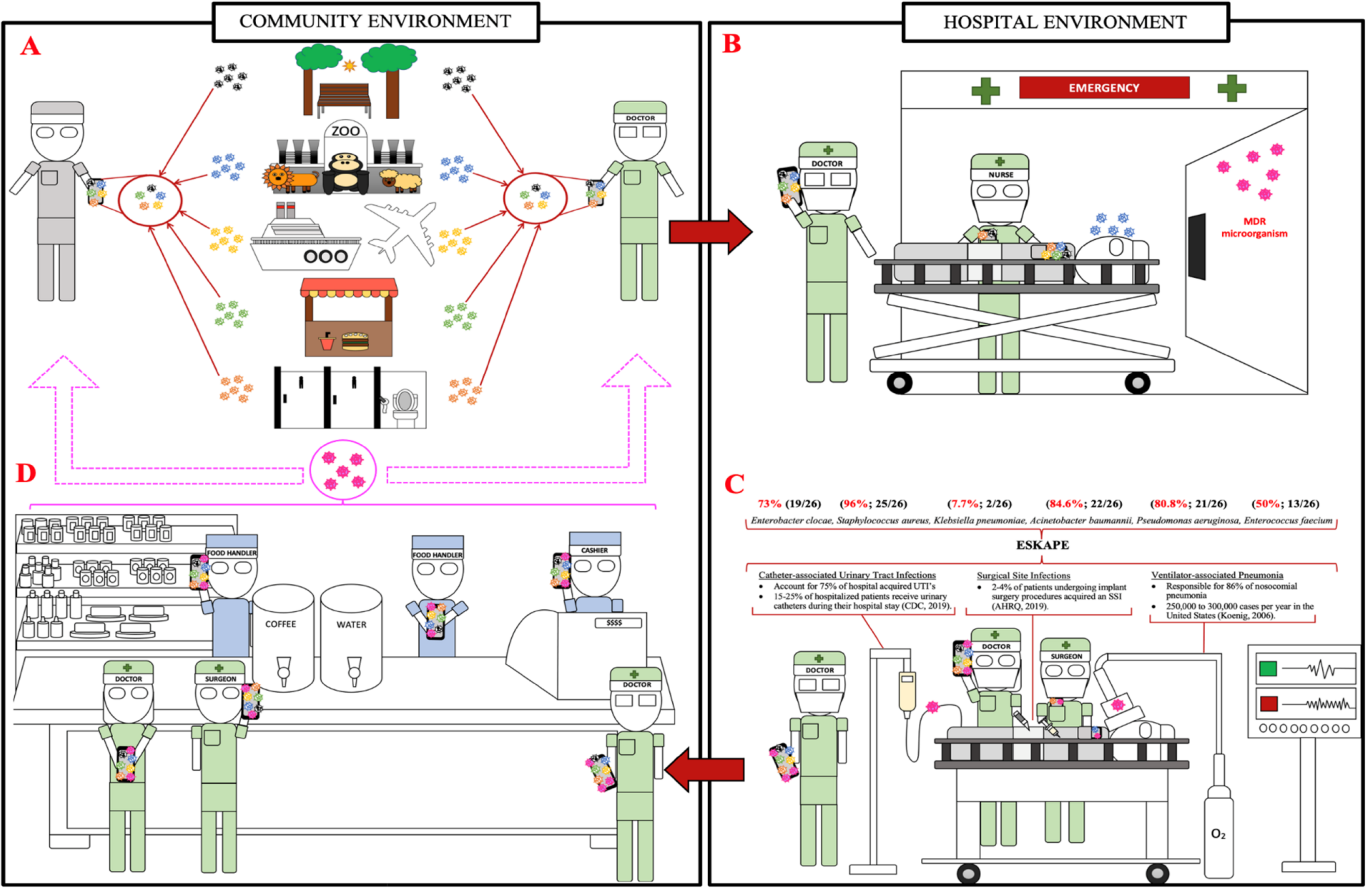
Contaminated mobile phones potential vectors of dissemination of germs in and out healthcare and community settings.

In hospitals, it is now commonplace for mobile phones to be used by the majority of HCW, they may however be counteracting the World Health Organisation hand hygiene campaigns. The efforts to limit exposure of microbes to patients may be nullified if mobile phones are not decontaminated regularly^19^. The number of microbes identified on phones does suggest that new measures of infection control in these vulnerable areas should be implemented. This should include mobile phone sanitisation as a corollary to the Five Moments of Hand Hygiene^20^. Mobile phones should now be considered as the ‘third hand’ from their users and subject to frequent decontaminations in hospitals (both health care staff and patients/visitors). An infographic shows the dissemination route of microbes derived from healthcare staff users and users of the community (Fig. 10).

Figure 10 illustrates the transmission dynamics of microbes derived from mobile phones and the possible inter-related dissemination of germs in and out healthcare and community settings. A. Mobile phones exposed to all sorts of community environments will harbour diverse microbes from the user’s hands. These organisms may persist on the surface of mobile phones and be a source of further downstream dissemination in other areas.

B. illustrates a patient of the community admitted at the hospital and both healthcare and patient’s mobile phones are contaminated. Germs in pink represent multi drug resistant nosocomial pathogens in hospitals.

C. on duty medical staff with their (non-sanitised) mobile phones might be the cause of nosocomial diseases contracted by vulnerable immuno-compromised patients during various procedures (ventilators, catheters, injections, open wound surgery.).

D. While nosocomial pathogenic and resistant microbes are present in hospitals, health care workers on duty might acquire such pathogens on the surface of their phones. At lunch or at the end of their shift medical professionals may disseminate these pathogens in the community.

## Author’s recommendation

This direct swab to metagenomic analysis study has revealed that hospital derived mobile phones used by health care workers, are accommodating niches for large amount of diverse pathogenic germs that are equipped with an arsenal of virulence genes and large spectrum of antibiotic resistance.

While this study took place in a hospital, the research highlights the need for the scientific community and public health authorities to further investigate the role mobile phones play as fomites. The potential for them to be vehicles for transmission and propagation of infectious microbes across health care settings needs to be addressed. Additionally, mobile phones harbouring a plethora of viable microbes are in circulation, with billions currently owned globally, and may be the means to establish, maintain or spread epidemics and pandemics. As an example, SARS-CoV-2 was detected on mobile phones and shown to survive on such platforms up to 28 days^21^. Undetected introductions of biothreats and invasive pathological organisms might be due to the billions of passengers travelling around the globe with ‘uncleaned’ mobile phones. Presence of SARS-CoV-2 omicron or delta variants on mobile phones need to be investigated.

Additionally, this research emphasises that the density of microbes found on mobile phones may be the ideal platforms for horizontal genetic transfers to occur among different species of micro-organisms such as transformation, conjugation, and transduction. Mobile phones may act as platforms for microbial multiplication and as a dynamic training ‘school’ for superbugs to evolve (and disseminate).

Mobile phones are dynamically contaminated with all sorts of microbes touched by the hands of their users thousands of times a day, even while in bathrooms. Mobile phones therefore have become our third hand. They are ‘dirty’ as are infrequently cleaned/sanitised and are completely negating first the worldwide gold standard hygienic hand washing practices and secondly the cost-effective public health and biosecurity prophylactic measures. Mitigation resides in sanitising mobile phones as frequently as we wash our hands with the adoption of new technology driven solution a like safety-certified enclosed ultraviolet-C emitting mobile phone sanitisers to clean phones in 10–20 s. This fast and efficient technology driven sanitisation of phones is practical as could be performed while health care workers practise hand hygiene. Presence in healthcare facilities of stations that can decontaminate both hands and mobile phones will prevent the risks of cross contamination and should be implemented in the five moments of hand washing.

**Figure 11:**
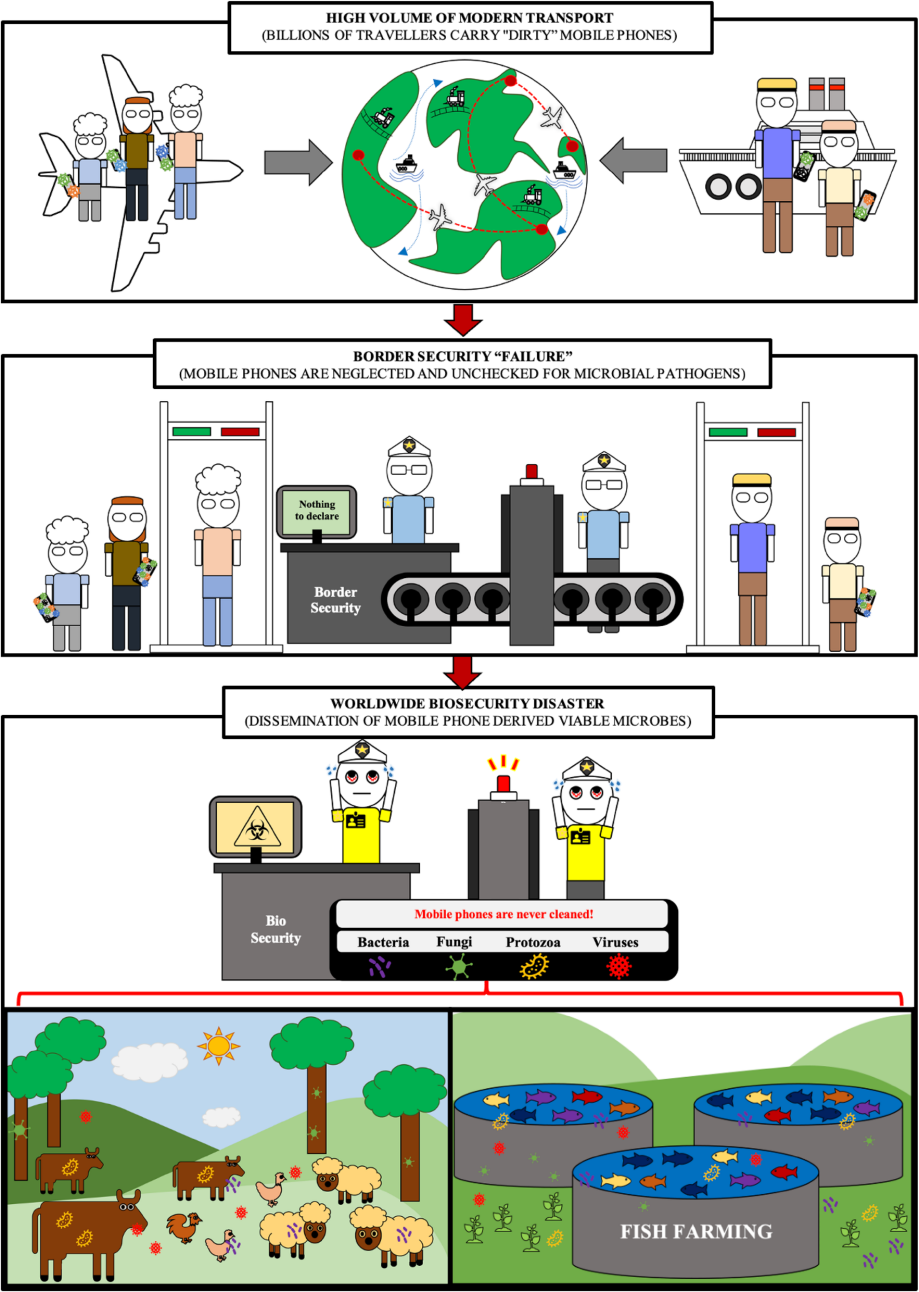
Mobile phone contaminated with microbes pose national and global biosecurity threats.

It also sends a strong message to the general community to prevent further global microbial dissemination. These metagenomics analysis findings revealed a real biosecurity concern with possible economically important disease repercussions that authorities must take seriously. Not only were some microbes on mobile phones highly resistant to multiple antibiotics, but cancer related viruses such as herpes viruses, polyomaviruses and human papillomaviruses are also of high concern for public health if mobile phones are not decontaminated in a daily basis. With 134 different antibiotic resistance genes and 419 different virulent factor genes found across all 26 mobile phones, the United Nation sustainable development goal number #3 ‘Good health and well-being’, is in peril. SDG#3 will undoubtfully fail to reach that goal by 2030 because of multiple factors that include: (i) a discovery void era of new antibiotics, (ii) paucity of research for alternative antimicrobial solutions and (iii) ‘third hand’ microbial laden mobile phones with multi drug resistant superbugs^22^. Hundreds of trillions of micro-organisms on the surface of billions of mobile phone fomites cross borders, by means of modern transport, un-noticed as Trojan horses. Custom security officers are not aware nor trained to prevent and stop the entry of these viable germs present on mobile phone. No measures or regulations exist in our hospitals or in our airports to decontaminate these mobile ‘petri-dishes harbouring in total impunity an array of pathogens. In the hands of billions of people mobile phones enter our health care settings, land in our countries and act as vectors to disseminating germs in all corners of the globe. Public Health and Biosecurity authorities should work ‘hands in hands’ to stop this silent ‘third hand’ driven pandemic and implement urgently regulations to actively decontaminate mobile phones as niches and reservoirs of viable microbes. The consequences for national and global biosecurity are outlined in Fig. 11.

Figure 11 Passengers of modern transport are per billions and carry with them billions of phones. While traveling around the globe, passengers returning home or in holiday trips pass through the customs without officer’s awareness that mobile phones carry all sorts of pathogens (viruses, bacteria, fungi, and protists) and proper sanitisation logistics to clean phones. The entry of billions of pathogens (including probably hard to control invasive germs) pass borders un-noticed and enter countries every day of the year. Downstream repercussions of un-controlled passage of all these viable microbes by means of trojan horse mobile phones are yet to be quantified in terms of economic losses due to inadequate biosecurity measures to decontaminate mobile phones at borders. Impacts on agriculture, native flora, marine fauna and native fauna as well as all livestock and aquatic farms from these invasive biothreats may be astronomical but yet not considered a national biosecurity priority.

### Supplementary data

Complete and extended of Figs. 8 and 9 heatmaps are available online as supplementary datasets (Supplementary Figs. 1 and 2).

Additionally, an excel sheet entitled “Supplementary data_Olsen et al-2022_Hits_abundance across all 26 samples“ is also available online as a supplementary data. That supplementary information provides details regarding the taxonomy Ids and abundance of microbes and genes found present or absent across the 26 samples investigated in that study.

## Supplementary Information

Supplementary Information 1.

Supplementary Information 2.

Supplementary Information 3.

## Data Availability

The sequencing fastq dataset files of all sequencing samples of this study are available and processed in the SRA database with the SRA BioProject accession number PRJNA828402 that can be available in Entrez (https://www.ncbi.nlm.nih.gov/sra/PRJNA828402). Each detailed accession number of the 26 datasets generated and analysed during the current study are available in the NCBI repository, PRJNA828402—SRA—NCBI(nih.gov).”
