# Supplementary figures and images for: Mobile phones are hazardous microbial platforms warranting robust public health and biosecurity protocols
Source: Sci Rep. 2022 Jun 15;12:10009. doi: 10.1038/s41598-022-14118-9 (PMC9199474; doi:10.1038/s41598-022-14118-9)

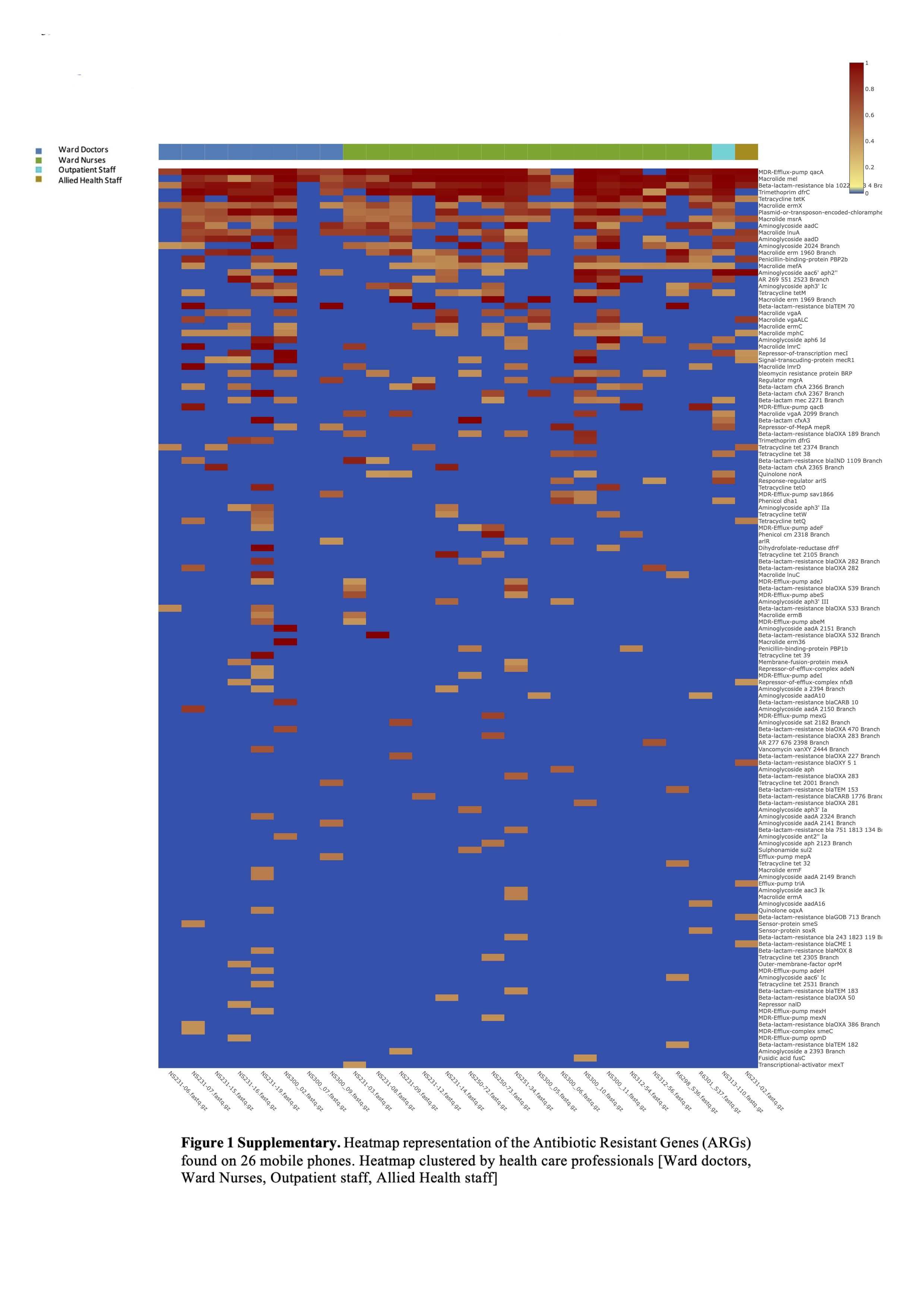

Supplement: Supplementary file 2 — Supplementary Information 2. [file 41598_2022_14118_MOESM2_ESM.jpg]

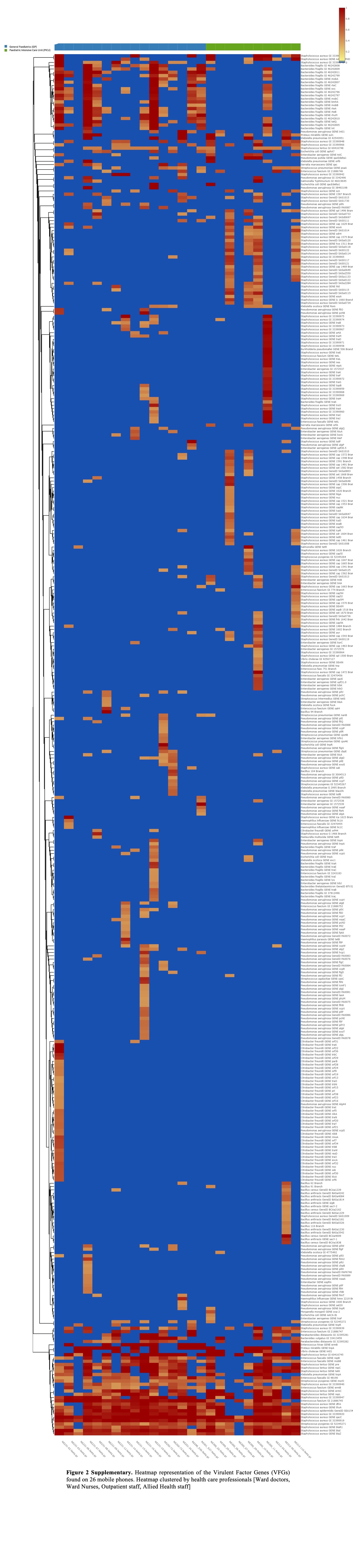

Supplement: Supplementary file 3 — Supplementary Information 3. [file 41598_2022_14118_MOESM3_ESM.jpg]
